# TYK2 correlates with immune infiltration: A prognostic marker for head and neck squamous cell carcinoma

**DOI:** 10.3389/fgene.2022.1081519

**Published:** 2022-12-01

**Authors:** Yaodong He, Yunshan Li, Junwei Xiang, Xu Huang, Mingyu Zhao, Yuanyin Wang, Ran Chen

**Affiliations:** Key Lab. of Oral Diseases Research of Anhui Province, College and Hospital of Stomatology, Anhui Medical University, Hefei, China

**Keywords:** TYK2, HNSCC, immune infiltration, prognosis, TCGA

## Abstract

Tyrosine kinase 2 (TYK2) is a member of the Janus kinase (JAK) family and is involved in immune and inflammatory signaling. TYK2 is overexpressed in several types of cancers and promotes the invasion and proliferation of cancer cells. Nevertheless, the roles of TYK2 in the prognosis and immune infiltration of head and neck squamous cell carcinoma (HNSCC) remain to be elucidated. In this study, the expression of TYK2 in HNSCC was evaluated based on the data retrieved from multiple databases and quantitative real-time polymerase chain reaction (qRT-PCR) analysis. The prognostic potential of TYK2 in patients with HNSCC was analyzed by Kaplan-Meier curves and Cox regression analysis. A TYK2-related risk assessment model was subsequently constructed by Least Absolute Shrinkage and Selection Operator (LASSO) regression analysis and stepwise multivariate Cox regression analysis. The association between the expression of TYK2 and the tumor immune microenvironment, immune checkpoints, and drug sensitivity was explored various packages in R. Cell function assays were finally used for exploring the effects of TYK2 on the growth and metastasis of HNSCC tumors. The expression of TYK2 was significantly upregulated in HNSCC and was found to be closely correlated with HPV status, gender, clinical grade, and TP53 mutation status. Survival analysis suggested that TYK2 is associated with better survival outcomes and acts as an independent prognostic indicator of HNSCC. The model constructed herein also performed well in terms of predicting patient prognosis. The expression of TYK2 was positively associated with the population of tumor-infiltrating immune cells, expression of immune checkpoint genes, and antitumor drug susceptibility. Functionally, TYK2 knockdown significantly promoted the proliferation, migration, and invasion of HNSCC cell lines *in vitro*. The findings demonstrated that TYK2 could serve as a suppressor of tumor growth and holds significant promise as a novel biomarker for assessing the prognosis of patients with HNSCC and aid in immunotherapy against HNSCC.

## Introduction

Head and neck squamous cell carcinoma (HNSCC) primarily includes cancers of the hypopharynx, nasopharynx, larynx, oropharynx, and the oral cavity (Johnson et al., 2020). As of 2020, the mortality rate of this disease was over 50% and approximately 930,000 patients were diagnosed with HNSCC (Sung et al., 2021). Despite continuous advances in radiotherapy, chemotherapy, and surgery, the rate of overall survival of patients with HNSCC remains low (Cramer et al., 2019a; Qin et al., 2021). Furthermore, the traditional methods for staging based on pathological features have a limited ability to assess patient prognosis and clinical treatment (Razzouk, 2014). Notably, it has been reported that the application of immunotherapy significantly improves the overall survival and quality of life of patients with HNSCC, especially in patients positive for HPV, PD-L1, or PD-1 (Cramer et al., 2019b; Galvis et al., 2020). Therefore, the identification of effective biomarkers is vital for the prognosis and development of immunotherapy against HNSCC.

Tyrosine kinase 2 (TYK2) is the first member of the Janus kinase (JAK) subfamily and acts as an intermediary between cytokine receptors and STAT transcription factors (Karjalainen et al., 2020). TYK2 is critical for the production of interferons and response to cytokines, and plays a vital role in innate immunity and inflammation, including inflammatory conditions resulting from viral infections and autoimmune diseases (Ubel et al., 2013). Numerous recent studies have indicated that TYK2 acts as an oncogene and promotes the migration, invasion, and metastasis of various cancers, including gastric, liver, colon, prostate, and lung cancers (Borcherding et al., 2021). However, one study reported that patients with lung adenocarcinoma who have high expression levels of TYK2 exhibit satisfactory prognosis (He et al., 2022). Previous studies have also demonstrated that TYK2 inhibits the growth and metastasis of breast cancer (Zhang et al., 2011). These findings imply that TYK2 plays a complex role in malignant tumors, and the role and mechanism underlying the effects of TYK2 in HNSCC remain to be elucidated.

TYK2 is essential for the cytotoxic functions of CD8^+^ T cells and natural killer (NK) cells, and promotes the maturation, cytotoxicity, and anti-tumor activity of NK cells in the host environment (Simonovic et al., 2019). A previous study demonstrated that TYK2-deficient CD8^+^ T lymphocytes lack cytotoxic activity (Simma et al., 2009). Additionally, TYK2 regulates the apoptosis of B cells *via* interferon-α (IFN-α), and its deficiency can induce the development of B cell lymphoma (Ubel et al., 2013). Another study demonstrated that tumor growth and metastasis was enhanced in TYK2-deficient mice owing to the activation of myeloid-derived suppressor cells (MDSCCs) (Zhang et al., 2011). The immune component of the tumor microenvironment (TME) of HNSCC consists of T cells, B cells, NK cells, macrophages, neutrophils, and MDSCCs (Johnson et al., 2020). In view of previous research results we speculate that TYK2 can be used as a biomarker for HNSCC and may be involved in the immune infiltration of the HNSCC tumor microenvironment, in addition, TYK2 has an impact on the malignant biological characteristics of HNSCC cells.

By employing comprehensive bioinformatic analysis, this study aimed to explore the effect of TYK2 on immune infiltration and its prognostic value in HNSCC. To this end, the expression of TYK2 in normal and carcinoma tissues was first compared, and the correlation between the expression of TYK2 and the clinical characteristics of patients with HNSCC was assessed. The prognostic role of TYK2 in patients with HNSCC was evaluated and a TYK2-related risk model was subsequently established. The immune infiltration landscape of HNSCC was comprehensively analyzed using multiple algorithms. The effects of TYK2 on tumor growth and metastasis in HNSCC were finally determined by cell function assays. The results of this indicate that TYK2 can provide insights into the clinical guidance of patients with HNSCC.

## Materials and methods

### Data acquisition and gene expression analysis

The corresponding clinical information and RNA-seq data of HNSCC samples (n = 499) and normal samples (n = 47) were obtained from TCGA. The TCGA database, the largest database of cancer genetic information, currently includes 33 cancer types based on large-scale genome sequencing, and genomic, transcriptomic, epigenetic, proteomic and other omics data are available. The complete clinical data and TYK2 expression in 499 patients with HNSCC are provided in Supplementary Table S1. The TIMER2 (http://timer.cistrome.org/) webserver was used for analyzing the pan-cancer expression of TYK2 (Li et al., 2020). A total of three datasets (accession IDs: GSE12452, GSE25099, and GSE30784) were retrieved from the Gene Expression Omnibus (GEO) for determining the expression levels of TYK2 in patients with HNSCC. Both GSE12452 and GSE30784 were obtained from the GPL570 platform [HG-U133_Plus_2] Affymetrix Human Genome U133 Plus 2.0 Array. GSE12452 includes data from 31 nasopharyngeal carcinoma (NPC) samples and 10 normal samples, whereas GSE3078 includes data from 167 OSCC samples and 45 normal samples. GSE25099 was obtained from the GPL5175 platform [HuEx-1_0-st] Affymetrix Human Exon 1.0 ST Array and includes data from 57 OSCC samples and 22 normal samples. TYK2 expression data, obtained by immunohistochemical staining studies, were obtained from the Human Protein Atlas (HPA) (Uhlen et al., 2017). The Human Protein Atlas is an open access database mapping all the human proteins in organs, tissues, and cells and uses integration of various omics technologies (Ponten et al., 2008). In the HPA database, protein expression rank was assessed by the staining intensity (strong/moderate/weak/negative) and fraction of stained cells (>75%/25–75%/< 25%), including high (strong with >25%), medium (strong with <25%, or moderate with >25%), low (moderate with <25%, or weak with >25%), and not detected (weak or negative with <25%) (Uhlen et al., 2010). The expression of TYK2 in different clinical subgroups classified on the basis of TP53 mutation status, HPV infection status, nodal metastasis status, individual cancer stage, tumor grade, gender, age, and race was comprehensively analyzed using the UALCAN webserver (http://ualcan.path.uab.edu/analysis.html) (Chandrashekar et al., 2017).

### Analysis of prognostic value

The Kaplan-Meier survival curves of TYK2 were generated with the Kaplan-Meier plotter (https://kmplot.com/analysis/) and GEPIA2.0 (http://gepia2.cancer-pku.cn/) (Tang et al., 2019; Lanczky and Gyorffy, 2021). GEPIA2 (http://gepia2.cancer-pku.cn/#index) is an updated version of GEPIA. It can be used to analyse the RNA expression sequencing data of 9736 tumour samples and 8,587 normal samples in TCGA and GTEx (Tang et al., 2017). The survival role of TYK2 was further evaluated based on the clinical data retrieved from the GEO database (accession ID: GSE65858). Univariate and multivariate Cox regression analyses were performed for identifying whether TYK2 can be used as a prognostic predictor independent of age, gender, grade, and T and N stages.

### Identification of TYK2-related genes and functional enrichment analysis

The HNSCC cohort from TCGA was used for co-expression analysis of TYK2, with the LinkedOmics portal (http://www.linkedomics.org/) (Vasaikar et al., 2018). For correlation analysis, genes with |cor| > 0.4 and adjusted FDR <0.05 in the Pearson correlation test were considered to be co-expressed (Supplementary Table S2). The samples of HNSCC from TCGA were split into the low expression group and the high expression group based on the median expression value of TYK2. The limma package in R was used for identifying the genes that were differentially expressed between the two groups, using |logFC| > 1.0 and FDR <0.05 as threshold. The genes related to TYK2 were subjected to Kyoto Encyclopedia of Genes and Genomes (KEGG) and Gene Ontology (GO) enrichment analyses using the clusterProfiler package in R (Yu et al., 2012). The Gene Ontology Consortium was used to provide a comprehensive, dynamic, and controlled source for functional genomics (Ashburner et al., 2000), including molecular function (MF), biological process (BP), and cellular component (CC).

### Construction of risk assessment model

To obtain survival-related TYK2 genes, univariate Cox regression analysis was examined for the association between prognosis in patients with HNSCC and genes. Each gene with *p*-value < 0.05 was finally chosen as a candidate gene for further analysis. To avoid overfitting, Least Absolute Shrinkage and Selection Operator (LASSO) analysis was used, and the most appropriate prognostic gene was identified. Then, an optimized risk score was established by a multivariate Cox regression analysis. The risk score for patients with HNSCC was estimated as follows: Risk score = (where Xi is the risk factor and Yi is the expression level of each gene). We plotted receiver operating characteristic (ROC) curves for the model at 1, 2, and 3 years and assessed the values of the Acak Information Criterion (AIC) at every point of the 3-year ROC curve to identify low- or high-risk scores of Cutoff points. Kaplan-Meier survival analysis determined the difference between the two groups in terms of survival to confirm the validity of this cutoff. R tools were used to visualize each patient’s survival curves and risk scores. The survival ROC, survival, and survminer packages in R were used for evaluating the predictive ability of the model.

### Immune infiltration analysis

The level of immune infiltration in patients with HNSCC was determined using established methods, including CIBERSORT-ABS, EPIC, MCPCOUNTER, QUANTISEQ, TIMER, and XCELL, and the association between the infiltration of immune cells and the expression of TYK2 was subsequently evaluated (Newman et al., 2015; Becht et al., 2016; Aran et al., 2017; Li et al., 2017; Racle et al., 2017; Finotello et al., 2019). The ESTIMATE package in R was also used for evaluating the TME status of each HNSCC sample, and the results were presented as immune/stromal/ESTIMATE scores (Sturm et al., 2019). The ggpubr package in R was used to visualize the differences between the scores of the high and low expression groups. The differences between the immune function of these groups were additionally determined by single-sample Gene Set Enrichment Analysis (ssGSEA). The different immune pathways enriched in the high and low TYK2 expression groups were also determined based on the immune-related gene set, c7. immunesigdb_HALLMARK, using the GSVA package in R.

### Analysis of immune checkpoint genes and chemotherapeutic sensitivity

As immune checkpoint inhibitors play an essential role in immunotherapy, we explored the relationship between the expression of TYK2 and immune checkpoint genes using TIMER2. In order to evaluate the effect of TYK2 on the treatment of HNSCC in the clinical settings, the half-maximal inhibitory concentration (IC50) of chemotherapeutic drugs in patients with HNSCC was calculated using the pRRophetic package in R, based on the anticancer drug sensitivity information retrieved from the GDSC project (Yang et al., 2013; Geeleher et al., 2014). The differences between the chemotherapeutic sensitivity of the high and low TYK2 expression groups were determined by the Wilcoxon rank-sum test, and the findings were depicted with box plots. In addition, the NCI-60 cancer cell line from CellMiner was used to analyze the association between the expression of TYK2 and drug response (Reinhold et al., 2012).

### Patient tissue samples and cell lines

A total of 25 pairs of rapidly frozen HNSCC tissues and surrounding healthy tissues were obtained from the patients during operation. The samples obtained from the patients were untreated before the procedure, and the HNSCC tissues were validated by thorough pathologic analysis. The patients signed a written informed permission form, and the study was approved by the Medical Ethics Committee at the Affiliated Stomatological Hospital of Anhui Medical University. All the methods were conducted in accordance to the appropriate regulations and guidelines.

The HNSCC cell lines, SCC4, SCC9, CAL27, HN4, HN6, and human normal oral epithelial keratinocytes (HOK), were purchased from Ninth People’s Hospital Affiliated to Shanghai Jiaotong University School of Medicine, Shanghai, China. All the cell lines were subjected to STR profiling and tested for *mycoplasma* contamination every 3 months. The cell lines were cultured in DMEM (BI, Israel) supplemented with 10% fetal bovine serum (FBS; BI, Israel), 1% penicillin, and streptomycin (NCM, Suzhou, China), at a temperature of 37°C in a humidified incubator containing 5% CO2.

### RNA extraction and quantitative real-time polymerase chain reaction (qRT-PCR)

Total RNA was extracted from the tissues and cell lines using TRIzol reagent (Thermo Fisher Scientific, United States). The concentration and purity of the RNA were determined using a NanoDrop Spectrophotometer (Thermo Fisher Scientific, United States). The total RNA isolated from the cell lines and tissues was reverse transcribed using the Prime Script RT Master Mix from Takara (Cat. #RR047A). A CFX96 Touch Real-Time PCR Detection System (Bio-Rad, Hercules, CA, United States) was used for qRT-PCR, according to the manufacturer’s protocol. GAPDH was used as the internal reference, and the reaction was conducted in two steps. The conditions for PCR were as follows: pre-denaturation at 95°C for 30 s, denaturation at 95°C for 5 s, and 50 cycles of annealing/extension at 60°C for 30 s. The threshold cycle (Ct) approach was used for estimating gene expression, and the 2^−ΔΔCT^ method was used for calculating the relative expression levels. The primer sequences used for qRT-PCR are listed in Table 1.

**TABLE 1:** A list of primers used in this study.

Gene	Primer	Sequence (5′ to 3′)
GAPDH	Forward	CAG​GAG​GCA​TTG​CTG​ATG​AT
	Reverse	GAAGGCTGGGGCTCATTT
TYK2	Forward	GGG​CAA​GCA​TGA​GTT​TGT​GAA​TGA​C
	Reverse	GGA​GAG​CGA​GGT​GAC​AGA​GGT​G

### Cell transfection

A negative control (NC) oligonucleotide and short interfering RNAs (siRNAs) were used for targeting TYK2 (Supplementary Table S3). For the cell culture experiments, SCC9 and CAL27 cells were seeded in 6-well plates, and Liposome 2000 (Invitrogen, United States) was added to the cells along with the siRNAs or the controls (General, Anhui, China) in accordance with the manufacturer’s instructions at a final concentration of 50 nM.

### Cell counting kit-8 (CCK-8) proliferation assay

A total of 3,000 cells were inoculated in each of the 96-well plates, and 10 μl of CCK-8 reagent was added to the culture medium after 0, 24, 48, and 72 h of transfection. The cells were then incubated at 37°C for 1 h, and the absorbance was measured at 450 nm using a microplate reader.

### Colony formation assay

The transfected SCC9 and CAL27 cells were inoculated in 24-well plates at a density of 800 cells per well, and incubated at 37°C in an atmosphere of 5% CO2 for 2 weeks. The cells were washed with phosphate buffer solution (PBS), fixed with 4% paraformaldehyde for 30 min, and stained with 0.1% crystal violet solution for 10 min. The colonies were subsequently counted and analyzed.

### Wound healing assay

Eighty percent fusion was achieved following the transfer of the transfected SCC9 and CAL27 cells to a 12-well plate. The single cell layers were scraped with the tip of a 10 μl pipette and the cells were washed thrice with PBS for removing the cellular debris, following which fresh medium containing serum was added to the cells. Representative images of cell migration were captured at three different high magnification fields after 0 and 24 h of scratching. The width of the scratch was estimated using the ImageJ software.

### Transwell migration and invasion assays

In the migration assay, the transfected SCC9 and CAL27 cells were inoculated in the upper chamber of a Transwell system (BD Biosciences, San Jose, CA, United States), and the lower chamber was filled with 500 μl of medium containing 10% FBS. After 24 h of inoculation, the cells remaining on the surface of the filter membrane were gently wiped off with a cotton swab, and the cells that passed through the membrane were fixed with methanol and stained with crystal violet solution. The number of cells were counted using an inverted microscope from three randomly selected fields of view, including the center and periphery of the membrane. The filters used in the Transwell system for the invasion assay were coated with Matrigel (BD Biosciences, San Jose, CA, United States), and the protocol for the invasion assay was similar to that of the migration assay.

### Statistical analysis

Data pertaining to the expression levels of TYK2 in the non-tumor tissues and HNSCC tumor tissues were retrieved from the GEO database, and the differences in TYK2 expression between the non-tumor and HNSCC tissues were analyzed by the Wilcoxon rank-sum test. Univariate and multivariate analyses were employed for Cox regression analysis. Te correlation between TYK2 mRNA expression and the clinicopathological variables was evaluated by Pearson’s Chi-square test. TYK2 expression betweendiferent clinicopathological groups was evaluated by the “edgeR” package of R software using Quasi-likelihood F-test. The differences in the TME scores, expression of immune checkpoint genes, and IC50 values between the high and low TYK2 expression groups were analyzed by the Wilcoxon rank-sum test. The correlation coefficient between TYK2 expression and immune infiltration cell scores was further estimated by Pearson correlation analysis. All the above statistical analyses were performed using R software, version 4.1.1. *p* < 0.05 was considered to be statistically significant unless otherwise stated.

## Results

### High expression of TYK2 in HNSCC

**FIGURE 1:**
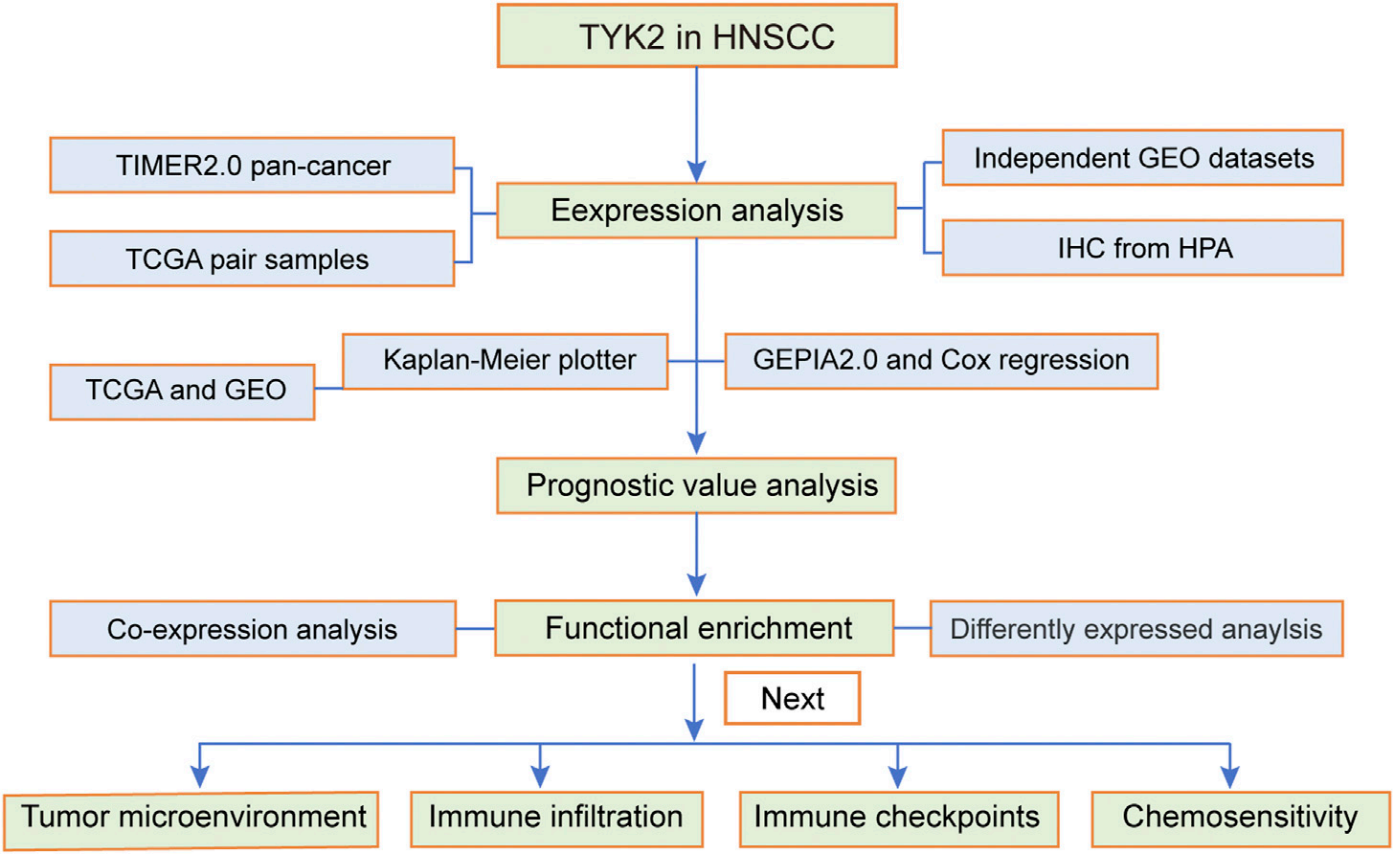
Flow chart of the present study. The TCGA-HNSCC cohort was used as the exploration set, and samples from GSE12452, GSE25099, GSE30784, and GSE65858 were used as the validation sets. HNSCC, head and neck squamous cell carcinoma.

**FIGURE 2:**
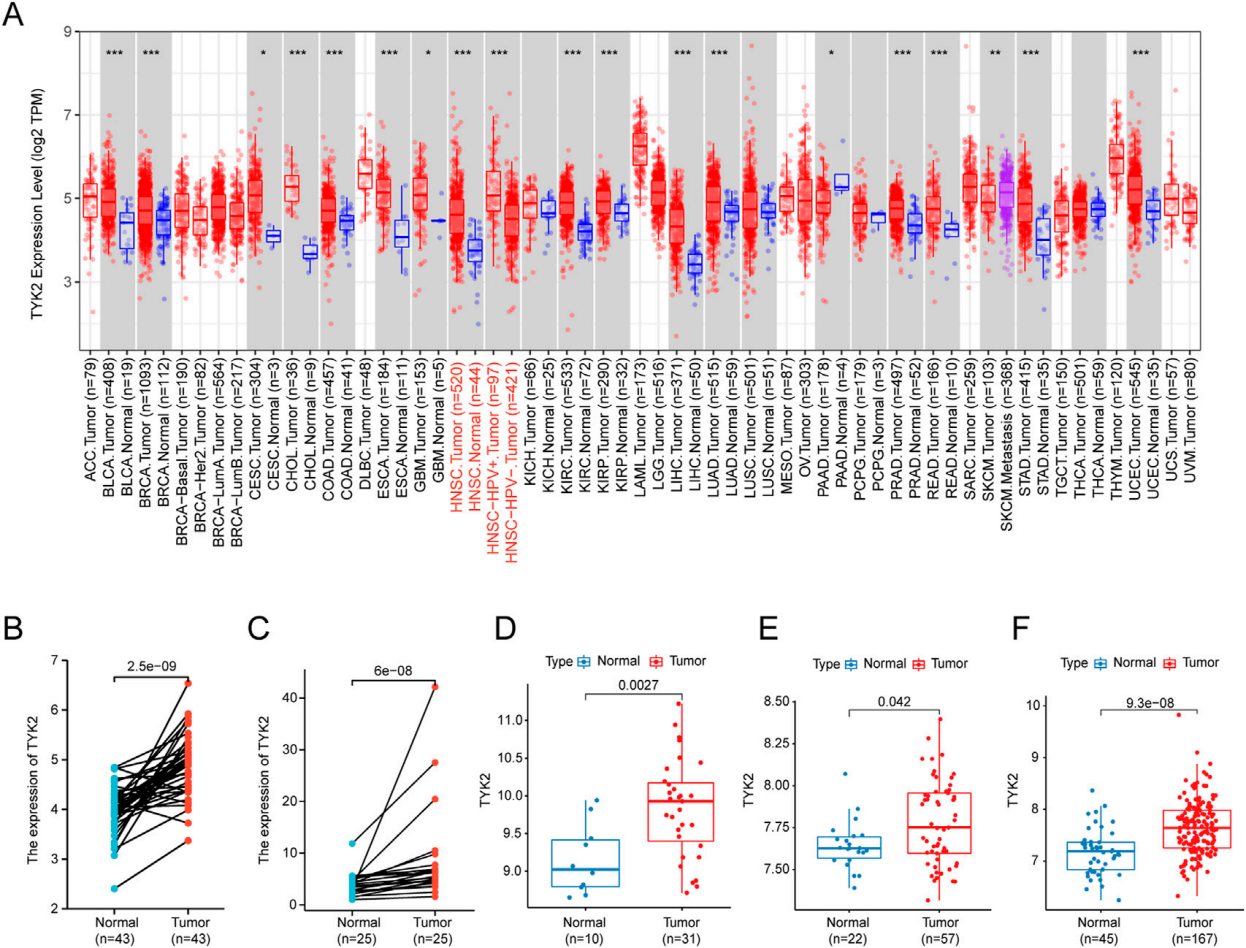
The expression profile of TYK2 in HNSCC. **(A)** High or low expression of TYK2 in various cancer tissues compared with normal tissues from the TCGA database. **(B)** The expression level of TYK2 was higher in HNSCC tissue than in the adjacent normal tissue in the TCGA database. **(C)** The relative expression of TYK2 in HNSCC tissues and adjacent normal tissues was detected by qRT-PCR (n = 25). **(D–F)** The expression level of TYK2 was more elevated in tumor tissues in GSE12452, GSE25099, and GSE30784 datasets.

The flow chart of the experiments performed in this study is presented in Figure 1. The expression levels of TYK2 mRNA in pan-cancer tissues were analyzed using the TIMER2 database, and the differential expression of TYK2 between the normal and tumor tissues was explored. As the results proved, TYK2 expression was higher in 17 TCGA tumors than in the corresponding normal tissues, including Bladder Urothelial Carcinoma (BLCA), Breast invasive carcinoma (BRCA), Cervical squamous cell carcinoma and endocervical adenocarcinoma (CESC), Cholangiocarcinoma (CHOL), Colon adenocarcinoma (COAD), Esophageal carcinoma (ESCA), Glioblastoma multiforme (GBM), HNSCC, Kidney renal clear cell carcinoma (KIRC), Kidney renal papillary cell carcinoma (KIRP), Liver hepatocellular carcinoma (LIHC), Lung adenocarcinoma (LUAD), Prostate adenocarcinoma (PRAD), Rectum adenocarcinoma (READ), Skin Cutaneous Melanoma (SKCM), Stomach adenocarcinoma (STAD), and Uterine Corpus Endometrial Carcinoma (UCEC). However, TYK2 expression was decreased in Pancreatic adenocarcinoma (PAAD) (Figure 2A). Notably, the expression of TYK2 was significantly higher in HPV + HNSCC tissues, compared to matched non-tumor tissues. Furthermore, the expression of TYK2 was upregulated in 43 HNSCC samples compared to paired adjacent samples (Figure 2B). In order to further verify whether the expression levels of TYK2 were upregulated in HNSCC tissues, we estimated whether the expression of TYK2 was upregulated in 25 HNSCC tissues relative to adjacent normal tissues by qRT-PCR. The results demonstrated that the expression of TYK2 mRNA was upregulated in the 25 HNSCC tissues compared to the adjacent non-tumor tissues, and were consistent with the data retrieved from the TCGA datasets (Figure 2C). Independent samples retrieved from GEO (accession IDs: GSE12452, GSE25099, and GSE30784) verified that TYK2 was overexpressed in HNSCC tissues (Figures 2D–F).

**FIGURE 3:**
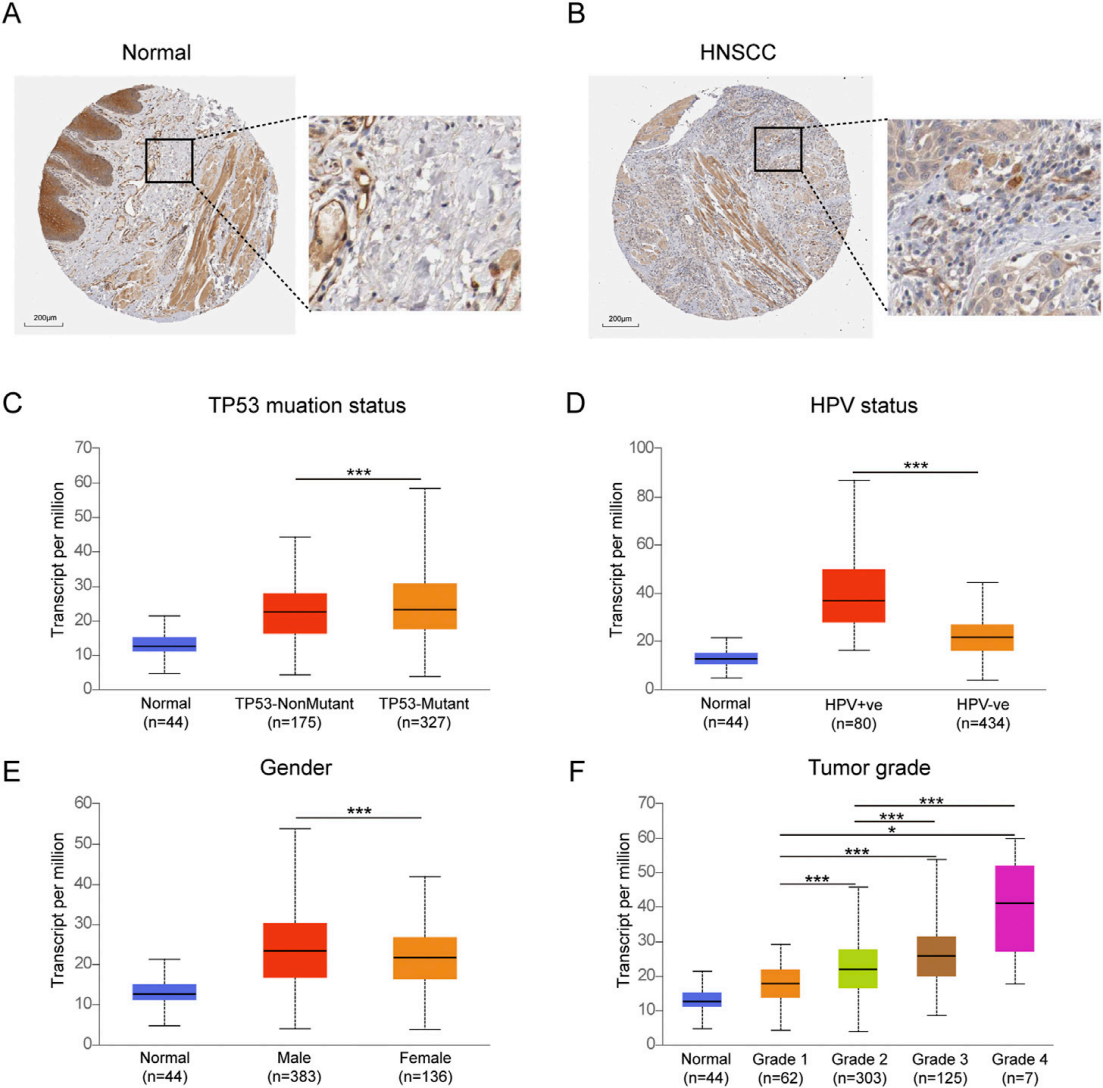
Immunohistochemical staining of TYK2 in normal oral tissues **(A)** and HNSCC tissues **(B)** from HPA. Comparison of TYK2 expression in different subgroups of TP53 status **(C)**, HPV status **(D)**, gender **(E)**, and tumor grade **(F)** (*p < 0.05, **p < 0.01, ***p < 0.001).

The expression of TYK2 protein was low in normal oral mucosal tissues, while the expression of TYK2 protein was high in HNSCC tissues, as depicted in Figures 3A, B. The clinicopathological variables of 499 primary HNSCC patients retrieved from TCGA were given in Table 2. Patients were classifed into two groups (low/high) according to their TYK2 expression level and based on the optimal cut-of value of OS calculated by the “survminer” package of R software. The differences in TYK2 expression between the clinical subgroups of HNSCC and the normal samples were assessed using the UNCLAN software. As shown in Figures 3C–F, the expression of TYK2 was significantly upregulated in the different subgroups of patients with HNSCC, including gender, HPV status, TP53 mutation status, and tumor grade subgroups, suggesting that TYK2 may serve as a potential biomarker for patients with HNSCC.

**TABLE 2:** Association between TYK2 expression and the clinicopathological variables in HNSCC patients (n = 499).

Variables	TYK2 expression		*p*
	High (n = 249)	Low (n = 250)	
**Age (year)**	—	—	0.227
>60	120 (24%)	135 (27.1%)	—
≤60	129 (25.9%)	115 (23%)	—
**Gender**	—	—	**0.046**
Female	56 (11.2%)	77 (15.4%)	—
Male	193 (38.7%)	173 (34.7%)	—
**Pathologic stage**	—	—	0.112
Stage I	13 (2.6%)	12 (2.4%)	—
Stage II	38 (7.6%)	31 (6.2%)	—
Stage III	32 (6.4%)	46 (9.2%)	—
Stage IV	124 (24.8%)	135 (27.1%)	—
unknow	42 (8.4%)	26 (5.2%)	—
**Histologic grade**	—	—	**< 0.001**
G1	19 (3.8%)	42 (8.4%)	
G2	134 (26.9%)	164 (32.9%)	—
G3	82 (16.4%)	37 (7.4%)	—
G4	2 (0.4%)	0 (0%)	—
unknow	12 (2.4%)	7 (1.4%)	—
**T stage**	—	—	0.296
T0	0 (0%)	1 (0.2%)	
T1	24 (4.8%)	21 (4.2%)	
T2	70 (14%)	61 (12.2%)	—
T3	40 (8%)	56 (11.2%)	—
T4	83 (16.6%)	88 (17.6%)	—
unknow	32 (6.4%)	23 (4.6%)	—
**N stage**	**—**	**—**	0.651
N0	90 (18%)	80 (16%)	—
N1	28 (5.6%)	37 (7.4%)	—
N2	79 (15.8%)	85 (17%)	—
N3	3 (0.6%)	4 (0.8%)	—
unknow	49 (9.8%)	44 (8.8%)	—
**M stage**	—	—	1.000
M0	93 (18.6%)	92 (18.4%)	—
M1	0 (0%)	1 (0.2%)	—
unknow	156 (31.3%)	157 (31.5%)	—
**Race**	—	—	0.500
African American	26 (5.2%)	21 (4.2%)	—
American Indian	1 (0.2%)	1 (0.2%)	—
Asian	3 (0.6%)	7 (1.4%)	—
Caucasian	210 (42.1%)	216 (43.3%)	—
Unknown	9 (1.8%)	5 (1%)	—

### Prognostic value of TYK2 in HNSCC

**FIGURE 4:**
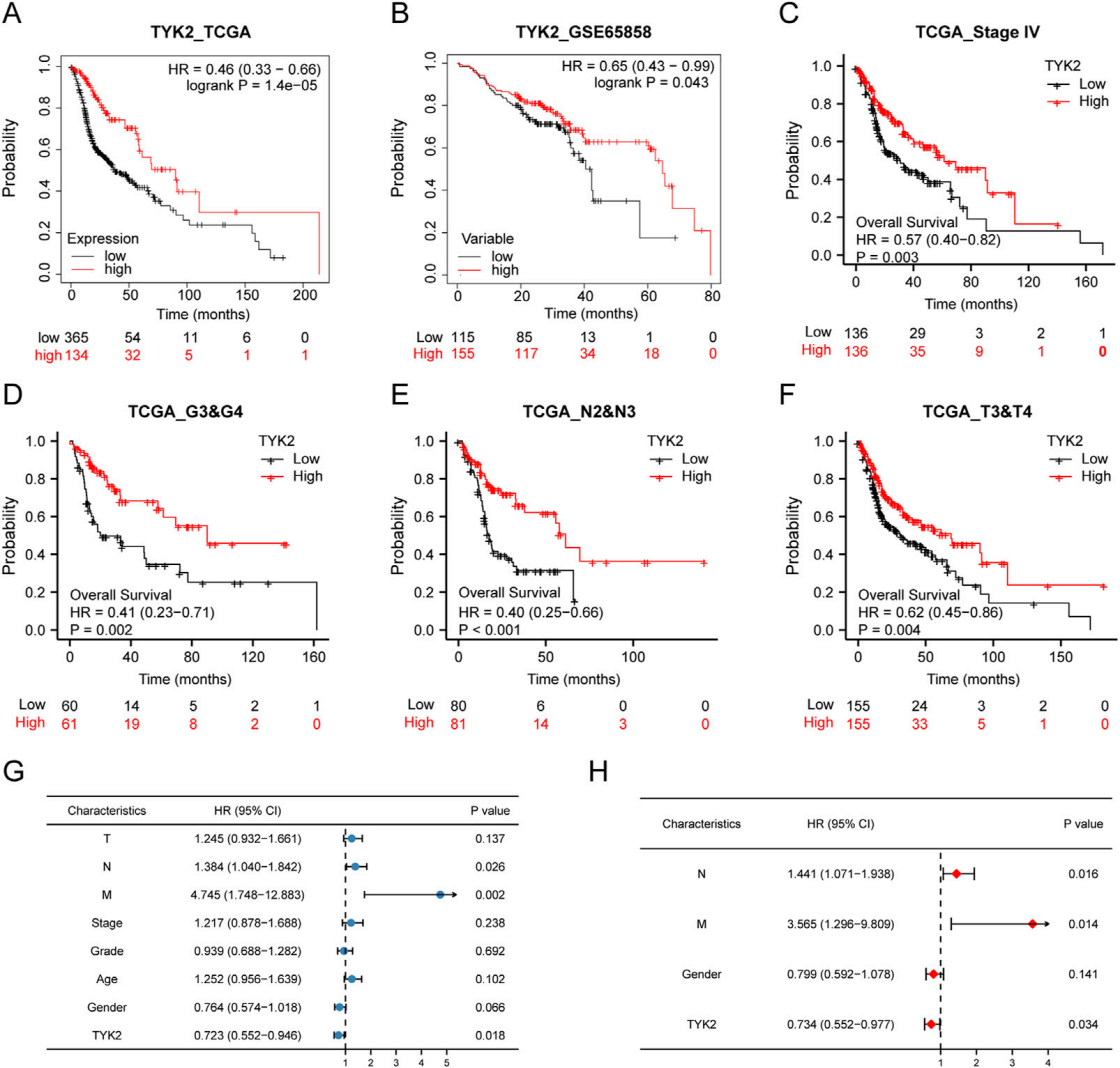
High-TYK2 expression was associated with a favorable prognosis in HNSCC samples. **(A)** and **(B)** Overall survival curves of TCGA and GEO patients by Kaplan-Meier plotter. **(C–F)** Overall survival curves of patients with advanced HNSCC. **(G)** and **(H)** Univariate and multivariate analyses of overall survival and clinicopathologic characteristics in TCGA patients.

The influence of TYK2 on the overall survival of patients with HNSCC was investigated using the Kaplan-Meier plotter. The survival curve indicated that the expression of TYK2 was obviously linked to the better prognosis of patients with HNSCC (HR = 0.46, *p* < 0.001) (Figure 4A). Further analyses revealed that these findings were consistent with the results obtained with GEPIA2.0, and patients with high TYK2 expression had better overall outcomes (HR = 0.61, *p* < 0.001) (Supplementary Figure S1). The clinical data retrieved from the GEO database (accession ID: GSE65858) further confirmed the above results (Figure 4B). Notably, the findings revealed that TYK2 is a vital prognostic protective factor in patients with advanced HNSCC (Figures 4C–F). Subsequent univariate Cox regression analysis revealed that N stage and TYK2 expression were significantly associated with the overall survival, and multivariate regression analysis further indicated that TYK2 could serve as a positive independent prognostic factor in patients with HNSCC (HR = 0.641, *p* = 0.004) (Figures 4G, H, Supplementary Table S4).

### Analysis of TYK2-related genes

**FIGURE 5:**
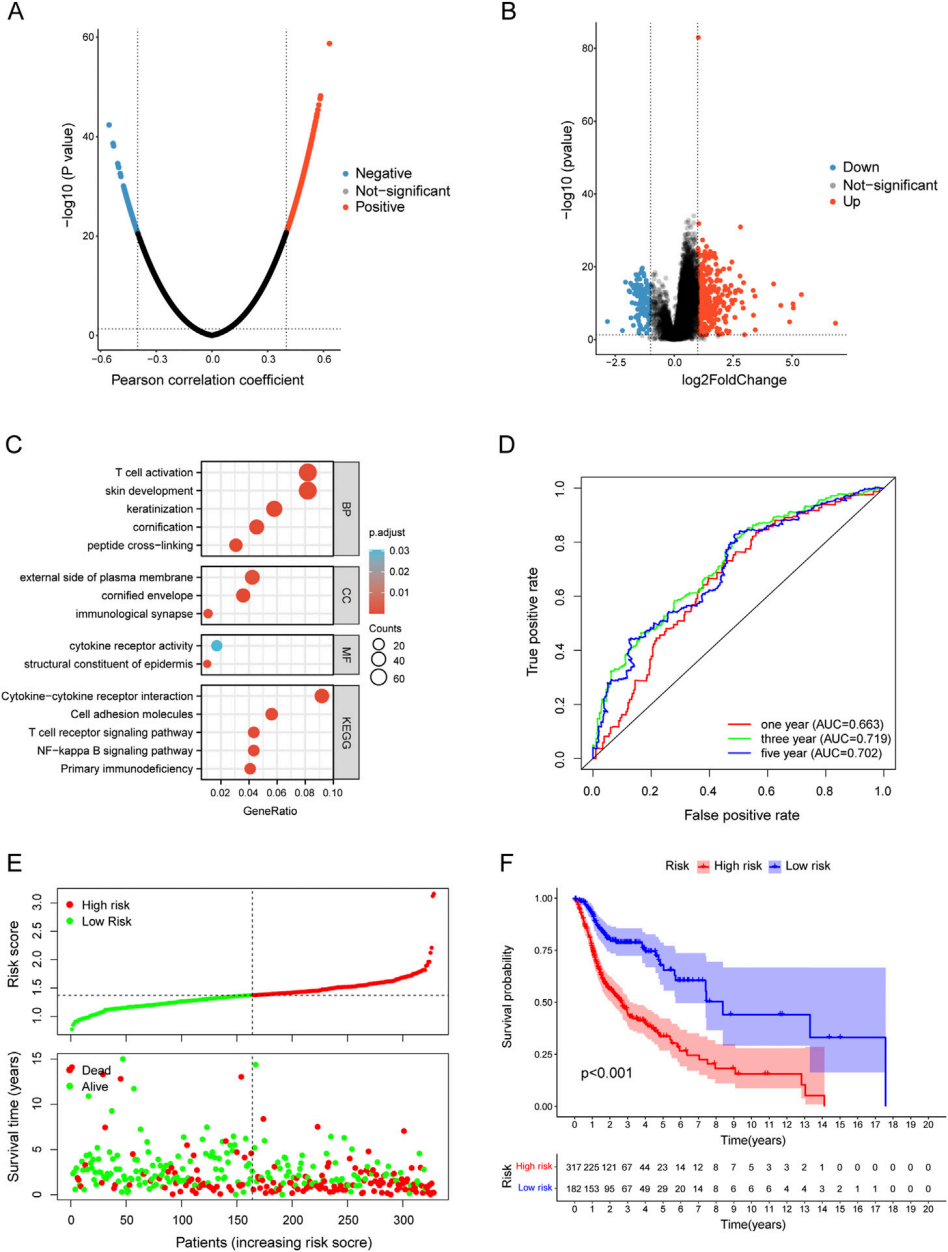
Functional enrichment of TYK2-related genes and conduction of TYK2-related risk model. **(A)** and **(B)**TYK2-related genes acquisition by Co-expression analysis and differential expression analysis. **(C)** GO and KEGG enrichment analysis of TYK2-genes. **(D)** The TYK2-related model exhibited good survival prediction ability. **(E)** and **(F)** High-risk groups had an unfavorable prognosis.

The LinkedOmics database was used for exploring the co-expression profile of TYK2 in HNSCC, and a total of 740 genes were found to be co-expressed with TYK2 (|cor| > 0.4, FDR <0.05) (Figure 5A). A total of 498 genes were found to be differentially expressed between the high and low expression groups (Figure 5B), and a total of 1087 TYK2-related genes were obtained after deleting the duplicated genes. These TYK2-related genes in HNSCC samples were then subjected to GO and KEGG enrichment analysis. In biological process (BP) category, these differentially expressed genes were mainly enriched in T cell activation and skin development, while in the cellular component (CC) category, these genes were primarily enriched in external side of plasma membrane. Molecular function (MF) enrichment results showed that these differentially expressed genes were mainly involved in cytokine receptor activity and structural constituent of epidermis. Furthermore, KEGG pathway enrichment analysis of these differentially expressed genes revealed the main enrichment in Cytokine−cytokine receptor interaction, T cell receptor signaling pathway and NF−kappa B signaling pathway (Figure 5C). These findings indicated that TYK2 might be involved in the immune response of HNSCC and affect the efficacy of immunotherapy *via* various mechanisms.

### Establishment and evaluation of TYK2-related risk model

A total of 201 survival-related TYK2 genes were initially identified in this study (Supplementary Table 5). Subsequent LASSO regression analysis was performed for screening the prognostic variables, and 17 TYK2-related genes were identified (Supplementary Figures S2A, B). The HNSCC risk assessment model was finally established from eight prognosis-related genes (Supplementary Figure S2C). The area under the predicted 1-year, 3-year, and 5-year survival curves were 0.663, 0.719, and 0.702, respectively, which indicated that the risk assessment model has adequate sensitivity for predicting survival (Figure 5D). Furthermore, we chose 0.929 as the cut-off point and divided patients into high-risk and low-risk groups, 254 patients were classified in the high-risk group and the remaining 244 patients were classified in the low-risk group (Supplementary Figure S2D). Figure 5E shows the RiskScores and survival rates for each case, indicating that patients in the low-risk group generally had better clinical outcomes than those in the high-risk group. Kaplan-Meier analysis and corresponding survival curves showed that patients with high-risk HNSCC had significantly shorter survival than those with low-risk (*p* < 0.001; Figure 5F).

### Relationship between TYK2 and tumor microenvironment

**FIGURE 6:**
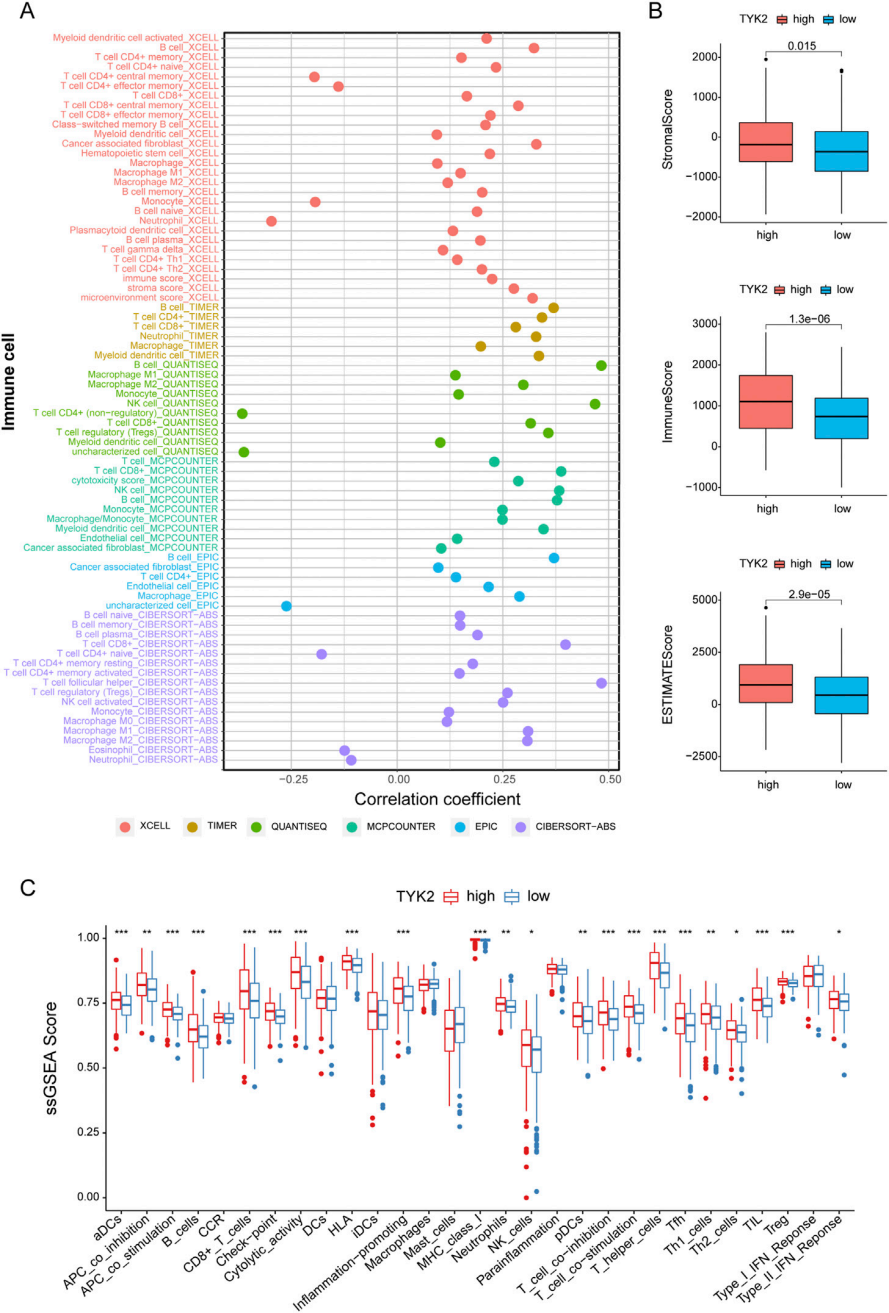
Estimation of immune-infiltrating cells. **(A)** The TYK2 expression was significantly positively correlated with most immune cells. The high-TYK2 group has higher TME scores **(B)** and more active immune function **(C)**.

In order to further elucidate the effect of TYK2 on the TME, we determined the infiltration of immune cells in patients with HNSCC. As can be seen from the results, TYK2 expression was positively related to most immune cells, including B cell (r = 0.483, *p* < 0.001), NK cell (r = 0.468, *p* < 0.001), CD8^+^ T cells (r = 0.398, *p* < 0.001), regulatory T cells (Tregs) (r = 0.358, *p* < 0.001), CD4^+^ T cells (r = 0.343, *p* < 0.001), cancer associated fibroblast (r = 0.329, *p* < 0.001) (Figure 6A, Supplementary Table S6). The results also demonstrated that the stromal score, immune score, and ESTIMATE score of the high TYK2 expression group were significantly higher than those of the low TYK2 expression group (Figure 6B). The results of ssGSEA further suggested that the immune response of patients with high TYK2 expression could be more active than that of patients with low TYK2 expression (Figure 6C). The heat map of the immune metabolic pathways revealed that the high TYK2 expression group was enriched in B cells, CD4, CD8, and other pathways that are upregulated in immune cells (Supplementary Figure S3).

### Prospects of TYK2 in the treatment of HNSCC

**FIGURE 7:**
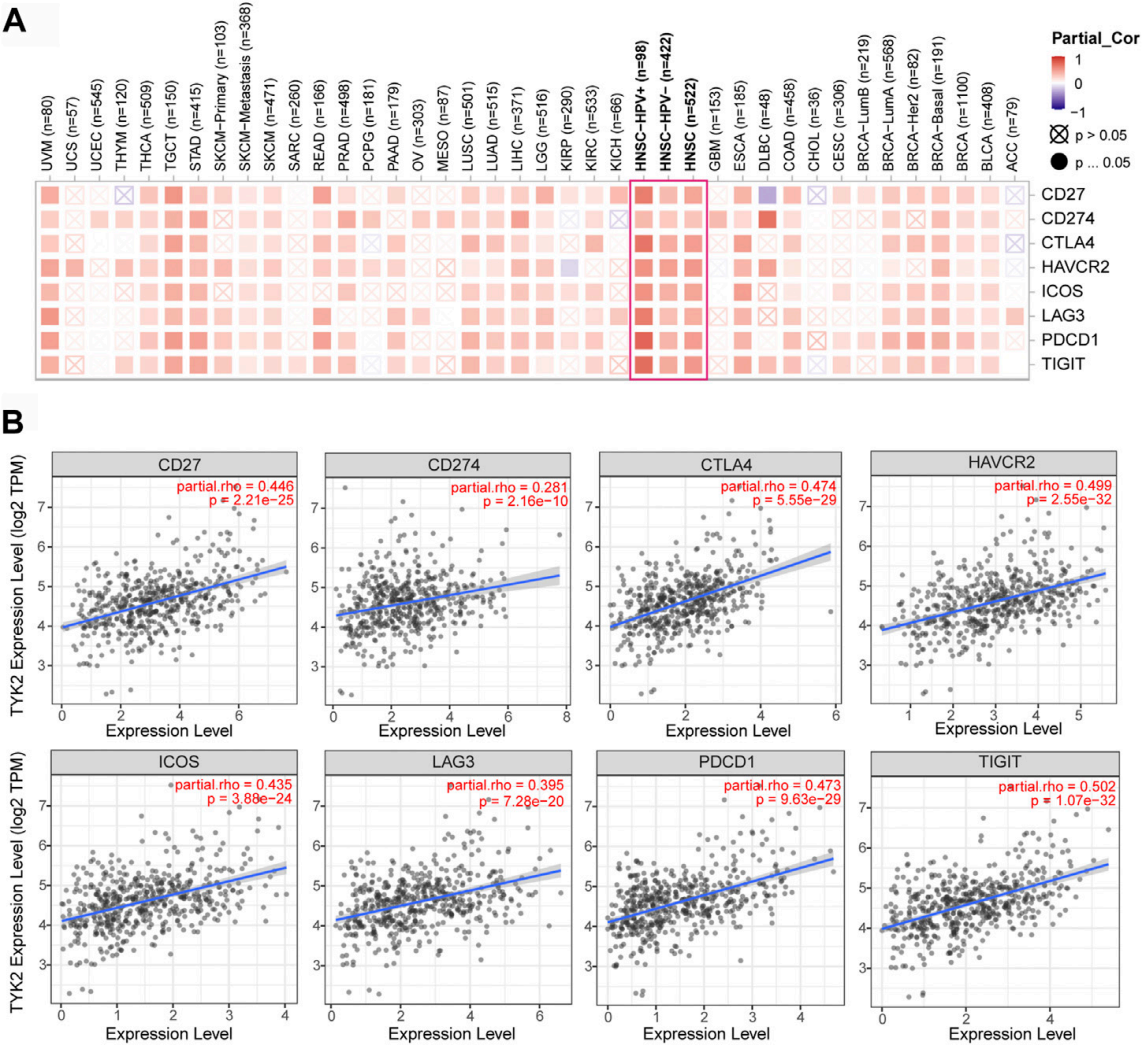
Correlation analysis between TYK2 expression and checkpoint-related genes in pan-cancer **(A)**. The TYK2 expression was significantly positively correlated with common immune checkpoints in HNSCC **(B)**.

The correlation between TYK2 expression and the expression of the main immune checkpoint genes, including IDO1, LAG3, PDCD1, CTLA4, HAVCR2, ICOS, CD27, CD274, and TIGIT, was determined using TIMER2. As depicted in Figures 7A, B, TYK2 expression was significantly positively correlated with the expression of the aforementioned immune checkpoint genes in HNSCC. Interestingly, the expression of TYK2 exhibited a stronger positive correlation with the expression of immune checkpoint genes, especially PDCD1 and TIGIT, in patients with HNSCC who were HPV-positive (Supplementary Figure S4).

**FIGURE 8:**
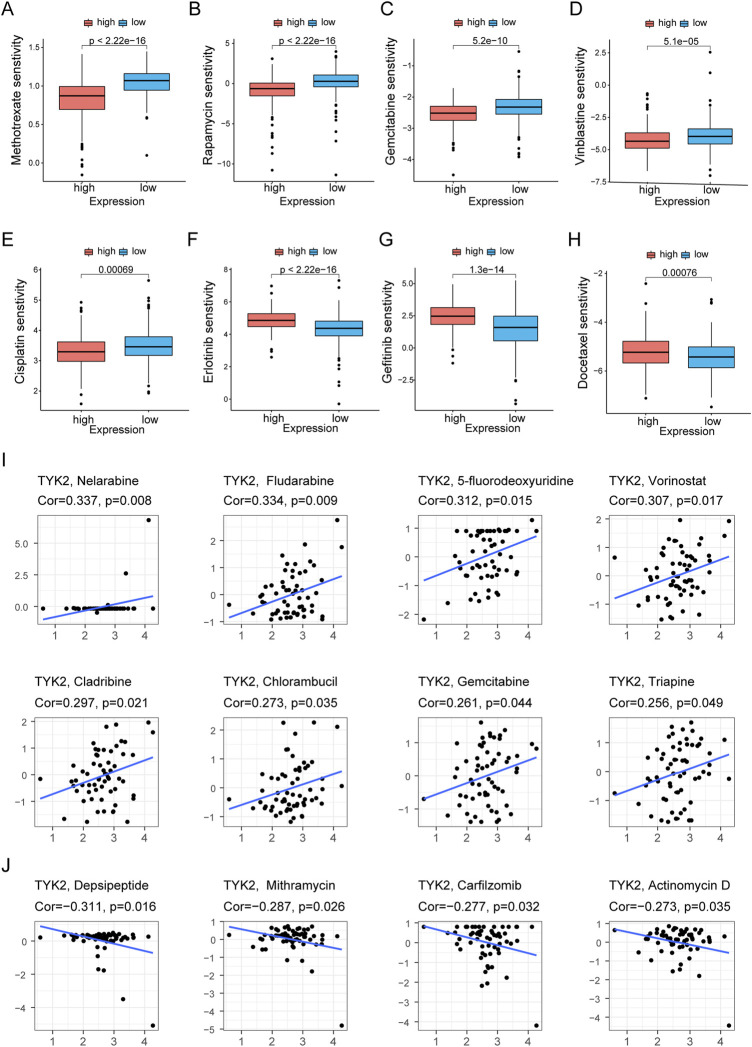
Differences of chemotherapeutic sensitivity in high- and low-TYK2 groups. The high-TYK2 group was related to lower IC50 of methotrexate, rapamycin, gemcitabine, vinblastine, cisplatin and paclitaxel **(A-E)**, while higher IC50 of erlotinib, gefitinib and docetaxel **(F-H)**. The relationship between TYK2 expression and potential anticancer drugs **(I,J)**.

The pRRophetic package in R was used to compare the differences in the sensitivity of the common chemotherapy drugs between the high and low TYK2 expression groups, and the findings revealed that the IC50 values of methotrexate, rapamycin, gemcitabine, vinblastine, cisplatin, and paclitaxel were lower in patients with high TYK2 expression (Figures 8A–E), while the IC50 values of erlotinib, gefitinib, and docetaxel were higher in these patients (Figures 8F–H). The data from CellMiner revealed that the expression of TYK2 was strongly associated with the sensitivity to 12 anticancer drugs. The expression of TYK2 was positively correlated to drug response in patients treated with eight drugs, including nelarabine, fludarabine, and 5-fluorodeoxyuridine (Figure 8I), but negatively correlated to the response to depsipeptide, mithramycin, carfilzomib, and actinomycin D (Figure 8J).

### TYK2 inhibited the proliferation, migration, and invasion of HNSCC cells *in vitro*

**FIGURE 9:**
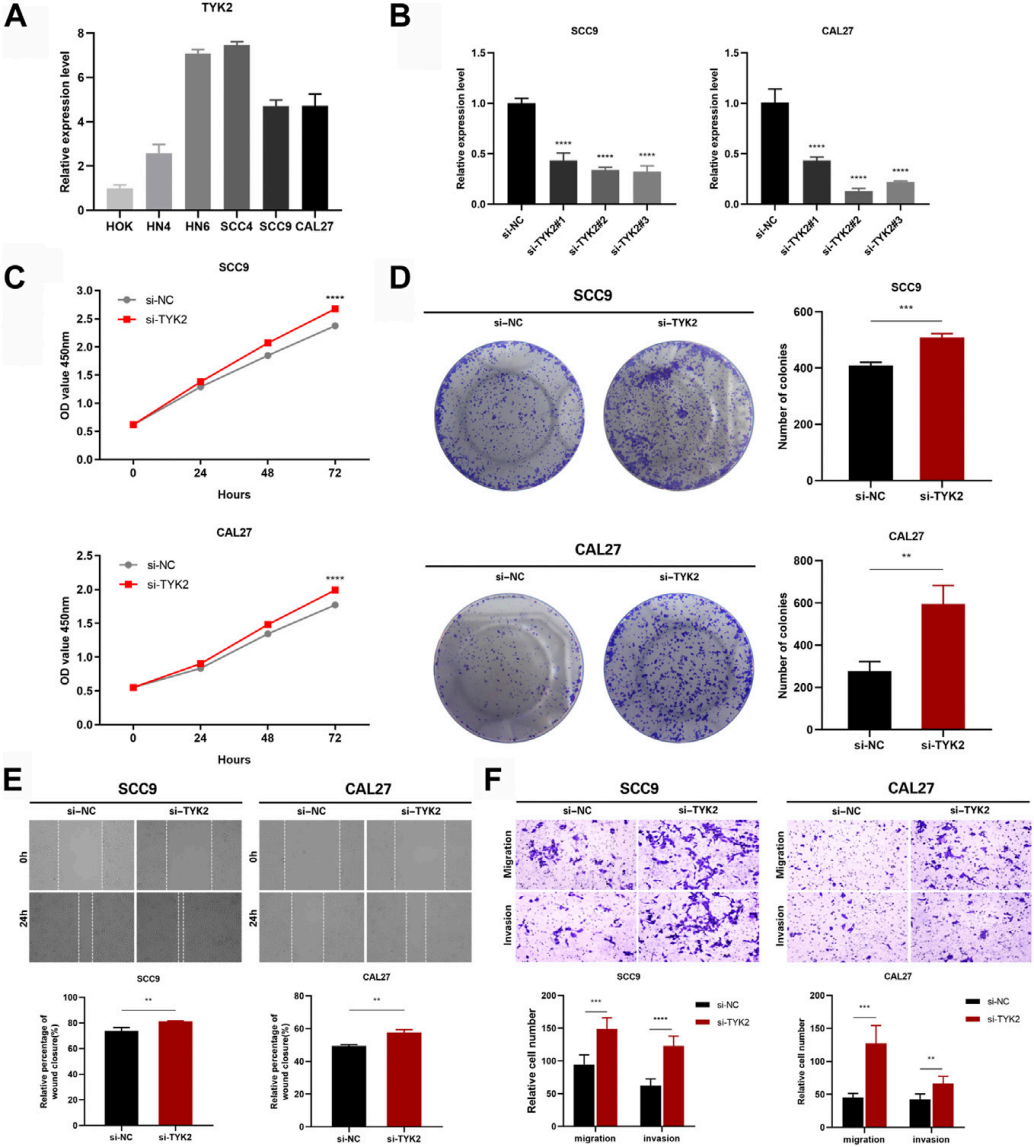
TYK2 inhibits the proliferation, migration and invasion of HNSCC cells *in vitro*. **(A)** The relative expression of TYK2 in the cells was detected by qRT-PCR. **(B)** qRT-PCR analysis of TYK2 expression in SCC9 and CAL27 cells treated with siRNAs. **(C)** and **(D)** The proliferation ability of SCC9 and CAL27 cells transfected with NC or si-TYK2 was examined by CCK-8 assay and colony formation assay. **(E)** and **(F)** The migration and invasion ability of the cells were detected by wound healing and transwell assays. Data were showed as mean ± SD,*p < 0.05, **p < 0.01, ***p < 0.001, ****p < 0.0001.

The expression of TYK2 in HOK and five HNSCC cell lines was identified by qRT-PCR, and the results revealed that the expression level of TYK2 in HOK cells was lower than that of the five HNSCC cell lines (Figure 9A). The SCC9 and CAL27 cell lines were finally selected as the representative HNSCC cells with TYK2 knockdown. The results of qRT-PCR revealed that the expression of TYK2 was markedly downregulated in SCC9 and CAL27 cells following treatment with siRNA fragments. Of the three siRNAs used for TYK2 knockdown, si-TYK2-2 was selected for further investigation owing to its superior silencing efficacy in SCC9 and CAL27 cells (Figure 9B). The results of the CCK-8 assay revealed that the downregulation of TYK2 markedly enhanced the proliferative activity of HNSCC cells (Figure 9C). Analysis of colony formation further revealed that the downregulation of TYK2 significantly enhanced the cloning ability of SCC9 and CAL27 cells (Figure 9D). The effects of TYK2 on the migration and invasion of HNSCC cells were investigated by wound healing and Transwell assays, respectively. The results demonstrated that the downregulation of TYK2 enhanced the invasion and migrating ability of SCC9 and CAL27 cells (Figures 9E, F). The findings of these experiments demonstrated that TYK2 could inhibit the proliferation, migration, and invasion of HNSCC cells.

## Discussion

HNSCC is a squamous cell tumor that can occur in any region in the head or neck (Johnson et al., 2020). Despite advances in the treatment of cancer, the overall survival rate for patients with HNSCC, especially at advanced stages, remains undesirable owing to the lack of effective and reliable prognostic biomarkers (Denaro et al., 2018). TYK2 is a ubiquitously expressed non-receptor protein tyrosine kinase that is generally associated with cell growth, development, and differentiation (Ubel et al., 2013). Accumulating evidence indicates that TYK2 functions as an oncogene in several malignancies and promotes carcinogenesis (Borcherding et al., 2021). In this study, we demonstrated that TYK2 is overexpressed in HNSCC tumor tissues and is closely associated with the clinical parameters, including TP53 mutation status, HPV status, and the grade and stage of cancer. TYK2 was also confirmed as a reliable and independent factor in assessing the prognosis of patients with HNSCC. Further analysis indicated that TYK2 may be involved in immune infiltration and was associated with common immune checkpoint genes. The study aimed to analyze the potential effects of TYK2 on the prognosis, immune infiltration profile, and treatment of patients with HNSCC.

In this study, we initially used the TIMER2 tool for analyzing the expression levels of TYK2 mRNA in pan-cancer samples. The results demonstrated that the expression of TYK2 mRNA was significantly upregulated in multiple cancers, including HNSCC, compared to that of normal matched non-tumor tissues. The differential expression of TYK2 in HNSCC was further validated by three independent datasets from the GEO database. In addition, survival analysis based on data from TCGA and GEO revealed that patients with high TYK2 expression had better survival rates. Univariate and multivariate Cox regression analyses demonstrated that TYK2 could be an independent protective factor for the prognosis of patients with HNSCC. The TYK2-related genes were subjected to GO and KEGG enrichment analyses for investigating the underlying mechanism and molecular functions of TYK2 in HNSCC. The results demonstrated that TYK2 might be involved in T cell activation and keratinization, and was associated with the risk factors for HNSCC, including primary immunodeficiency. The findings confirmed that TYK2 might play an active role in tumor immune surveillance and defense in HNSCC. A TYK2-related risk assessment model was finally constructed in this study, and the model was found to have good predictive potential in assessing the prognosis of patients with HNSCC.

Immune infiltrating cells play a vital role in the TME and strongly influence the response and prognosis of immunotherapy (Jochems and Schlom, 2011). In this study, we observed that the infiltration of B cells, CD4^+^ and CD8^+^ T cells, and macrophages was significantly positively related to the expression of TYK2, which indicated that the population of these immune cells is relatively higher in patients with high TYK2 expression. CD8^+^ T cells are vital effector cells in anti-tumor immunity (van der Leun et al., 2020), and the increased infiltration of CD4^+^ and CD8^+^ T cells in HNSCC tumor tissues is associated with favorable prognosis (de Ruiter et al., 2017). B cells are known to induce both tumor promotion and anti-tumor immunity in HNSCC (Lechner et al., 2019). It has been reported that macrophages are related to tumor growth and invasion and are particularly abundant in the TME of HNSCC (Kumar et al., 2019). We therefore hypothesize that the differences in the proportions and subtypes of immune infiltrating cells could be responsible for the differences in the survival of patients with high and low TYK2 expression. The findings also demonstrated that the group with high TYK2 expression had higher TME scores, suggesting that TYK2 may be involved in altering the TME of HNSCC.

The findings of this study also indicated that the expression of TYK2 was related to the expression of the common immune checkpoint genes, including CTLA4, CD274, PDCD1, and TIGIT. The finding suggested that immunotherapy could be more efficacious in the treatment of patients with high TYK2 expression. Notably, the expression of CTLA4, a classic T-cell response inhibitory checkpoint gene, was found to be upregulated in the high TYK2 expression group (Pardoll, 2012). The findings revealed that the CD8^+^ T cells were relatively enriched in the high TYK2 expression group; however, the overexpression of immune checkpoint genes, such as CTLA4, inhibited the cytotoxic function of CD8^+^ T cells. The anti-CTLA4 drug, ipilimumab, could therefore serve as an effective therapeutic agent in patients with high TYK2 expression (Jie et al., 2015). Similarly, another canonical immune checkpoint gene, PDCD1, was upregulated in patients with high TYK2 expression (Topalian et al., 2012). We therefore speculated that the anti-PDCD1 drug, nivolumab, could be applied clinically for these patients (Saada-Bouzid et al., 2017). Notably, the results demonstrated that the expression of TYK2 was more strongly correlated with the expression of the immune checkpoint genes, including PDCD1 (cor = 0.744) and TIGIT (cor = 0.705), in patients with HNSCC who were HPV-positive. Therefore, immunotherapy could be more effective for patients with HNSCC who are HPV-positive and have high TYK2 expression. The findings of this study also revealed that patients with high TYK2 expression were more sensitive to cisplatin, methotrexate, and paclitaxel than patients with low TYK2 expression. Cisplatin is the standard chemotherapy regimen for patients with advanced HNSCC (T ≥ T3). However, platinum sensitivity could be not investigated in this study owing to the lack of a suitable chemotherapy cohort. We also identified several potential drugs, including fludarabine, vorinostat, and cladribine, for patients with HNSCC who have high TYK2 expression.

Preclinical studies on cell lines and mouse models have reported that the application of TYK2 inhibitors, including NDI-031301 and SAR-20351, induces tumor regression in T-cell acute lymphoblastic leukemia and solid cancers with significant survival benefits (Akahane et al., 2017). In addition, the inhibition of TYK2 reduces the aggressiveness of breast and esophageal cancer cells (Ide et al., 2008; Jia et al., 2021). The findings of these studies suggested that TYK2 inhibitors hold promise for targeted cancer therapy. However, the results of this study demonstrated that high TYK2 expression is a favorable prognostic factor in HNSCC; therefore, TYK2 inhibitors should be used with caution in patients with HNSCC.

The present study has certain limitations. First, the inhibitory effect of TYK2 on the malignant progression of HNSCC cells was only investigated *in vitro*. Therefore, the findings of this study need to be validated with animal models in future studies. Second, while previous studies have reported that TYK2 acts an oncogene, the results of this study suggested that TYK2 acts as a prognosis protective factor in HNSCC. Therefore, the mechanism of regulation of TYK2 expression and immune infiltration in HNSCC require further clarification. In addition, gene-based markers are also insufficient as prognostic models for predicting patient outcomes or as biometric features. It is necessary to build network or sub-network markers for predictions to be more meaningful and precise. In order to investigate the relationship between gene expression patterns, representative protein-protein interactions, and clinical metastatic potential, Song et al. (Song et al., 2015) developed a method to identify survival prognostic subnetwork signatures (SPNs), which has proven to be more accurate and effective in predicting survival time without distant metastases. Finally, the single-cell level needs to be addressed in order to address the variations in tumor immune microenvironment under various therapeutic regimens. According to reports, a single-cell multi-omics gene co-regulation method (SMGR) was created by combining single-cell RNA sequencing and sequencing of single-cell transposase-accessible chromatin to find cis-elements and regulatory networks in mixed phenotypic acute leukemia cells (Song et al., 2022). To test and understand the findings, more thorough models and integrative methodologies are required.

## Conclusion

In summary, the present study demonstrated that the expression of TYK2 is high in HNSCC tissues, and the high expression of TYK2 is related to the increased infiltration of immune cells and survival in patients with HNSCC. The findings also highlight the critical role of TYK2 in the development of HNSCC and its potential prognostic and therapeutic value.

## Data Availability

The datasets presented in this study can be found in online repositories. The names of the repository/repositories and accession number(s) can be found in the article/Supplementary Material.
