# Supplementary material for: TYK2 correlates with immune infiltration: A prognostic marker for head and neck squamous cell carcinoma
Source: Front Genet. 2022 Dec 1;13:1081519. doi: 10.3389/fgene.2022.1081519 (PMC9752815; doi:10.3389/fgene.2022.1081519)
Supplement: Supplementary file 3 [file Image4.pdf]

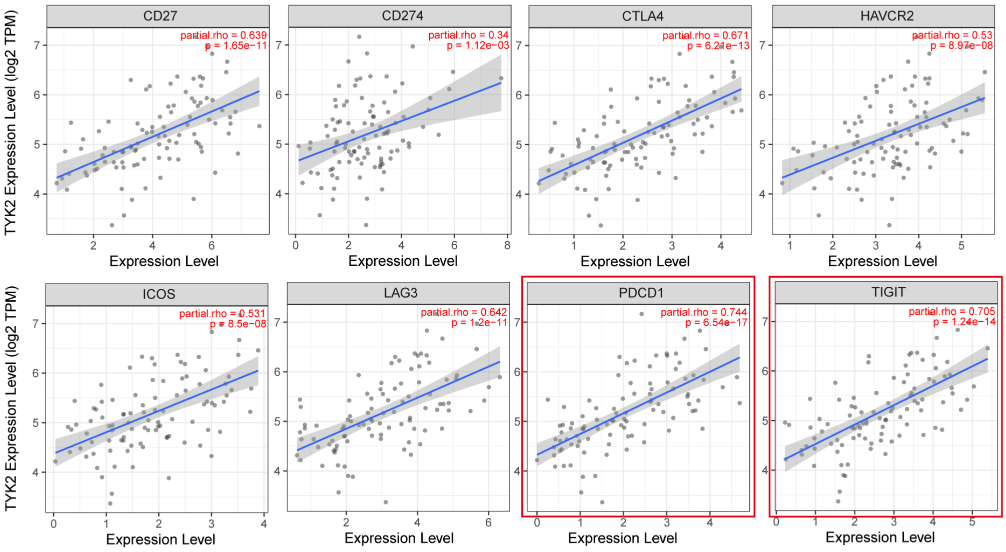

Figure S4. Correlation analysis between TYK2 expression and immune checkpoint genes in HPV-positive patients.
