# Supplementary material for: TYK2 correlates with immune infiltration: A prognostic marker for head and neck squamous cell carcinoma
Source: Front Genet. 2022 Dec 1;13:1081519. doi: 10.3389/fgene.2022.1081519 (PMC9752815; doi:10.3389/fgene.2022.1081519)
Supplement: Supplementary file 4 [file Image2.pdf]

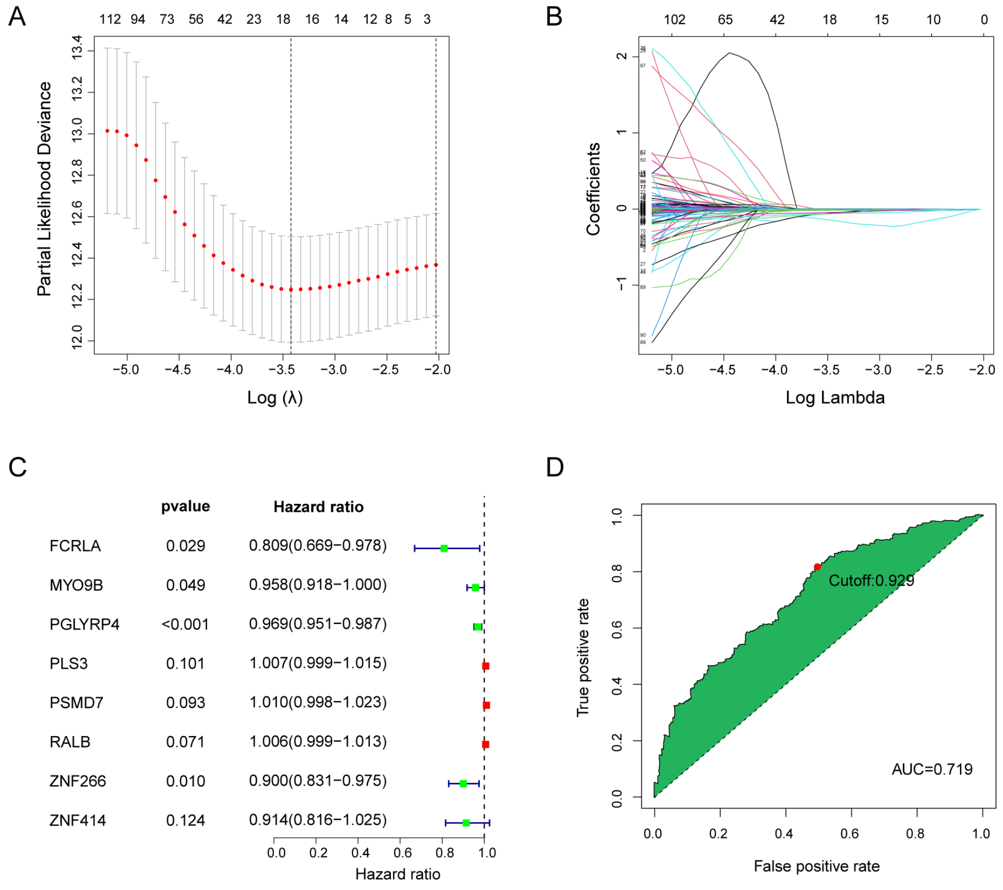

Figure S2. (A, B) LASSO regression of 41 TYK2-related genes. **(C)** Forest map of step multivariate Cox regression. **(D)** The maximum inflection point on the 3-year ROC curve was 0.929.
