# Supplementary figures and images for: TYK2 correlates with immune infiltration: A prognostic marker for head and neck squamous cell carcinoma
Source: Front Genet. 2022 Dec 1;13:1081519. doi: 10.3389/fgene.2022.1081519 (PMC9752815; doi:10.3389/fgene.2022.1081519)

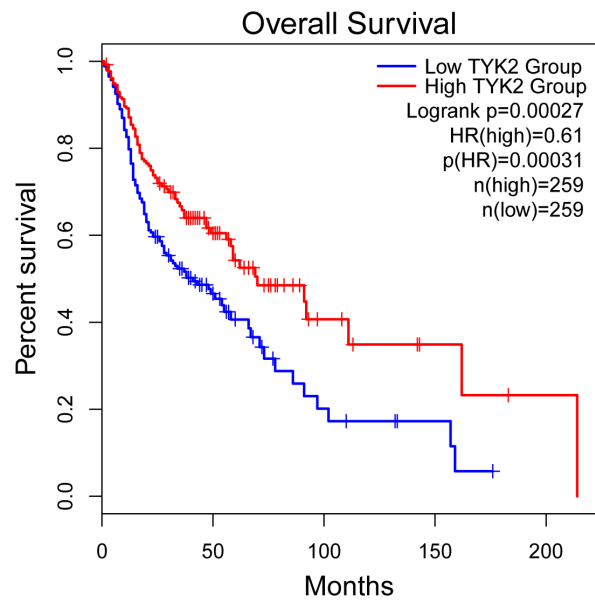

**Figure S1.** Overall survival curves of TCGA patients by GEPIA2.0.

Supplement: Supplementary file 8 [file Image1.pdf]
