# Supplementary material for: TYK2 correlates with immune infiltration: A prognostic marker for head and neck squamous cell carcinoma
Source: Front Genet. 2022 Dec 1;13:1081519. doi: 10.3389/fgene.2022.1081519 (PMC9752815; doi:10.3389/fgene.2022.1081519)
Supplement: Supplementary file 5 [file Image3.pdf]

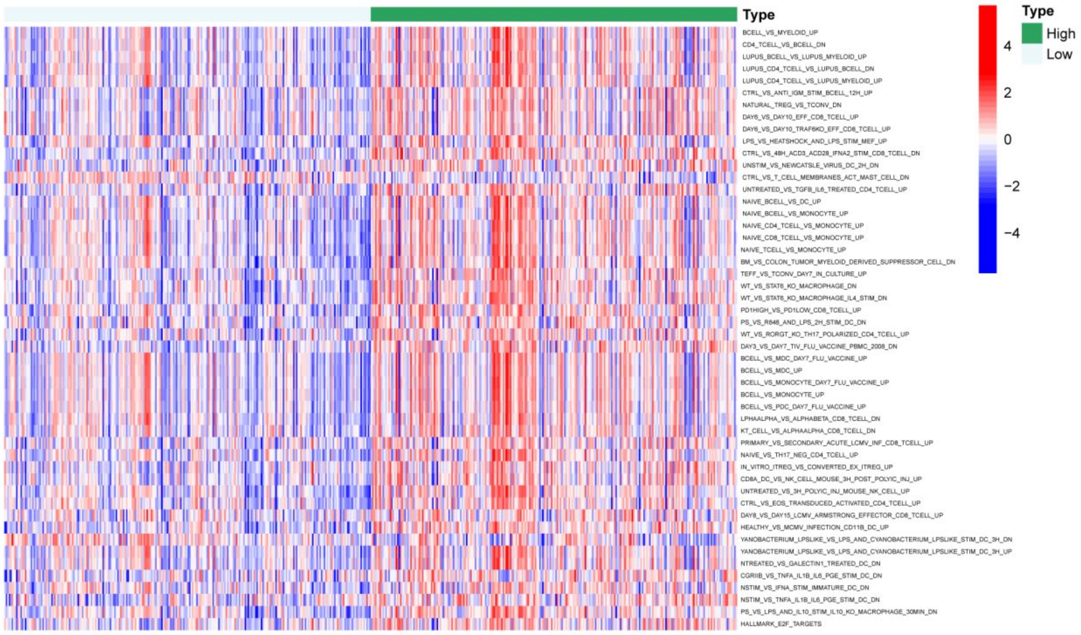

Figure S3. Heat map of immune pathway differences between high- and low-expression groups.
